# Comment on Kolivas et al. A 6-Month mHealth Low-Carbohydrate Dietary Intervention Ameliorates Glycaemic and Cardiometabolic Risk Profile in People with Type 2 Diabetes. *Nutrients* 2025, *17*, 937

**DOI:** 10.3390/nu18071114

**Published:** 2026-03-30

**Authors:** Jedha Dening

**Affiliations:** Independent Researcher, Brisbane 4000, Australia; j.dening@diabetesmealplans.com

Kolivas and co-authors reported the outcomes of a 6-month mHealth dietary intervention for people with type 2 diabetes (T2D) [1]. This is a valuable contribution to the limited evidence base for digital nutrition interventions, particularly in the Australian context. To ensure accurate interpretation and appropriate comparisons across the literature, several reporting issues warrant clarification or correction.

The article’s comparison to the T2Diet study randomised controlled trial (RCT) [2] misrepresents those findings. The authors reported the “T2Diet intervention group reduced HbA1c by 0.6%, body weight by 3.3 kg, and BMI by 1.1 kg/m^2^,” citing between-group differences. The published within-group reductions for the T2Diet intervention group were HbA1c 0.94%, body weight 4.36 kg, and BMI 1.48 kg/m^2^—all statistically significant [2]. This distinction matters: between-group differences reflect comparative effectiveness, whereas within-group changes reflect absolute participant response [3]. The comparison, as presented, overstates the relative effect of the single-arm intervention while understating the T2Diet RCT outcomes.

Similarly, the article appears to misinterpret data from the authors’ studies [1,4], potentially reflecting selective emphasis rather than analytic differences. The article claims significant weight improvement in prior work and over six months. However, neither the three-month (*p* = 0.200) [4] nor six-month (*p* = 0.10) [1] analyses demonstrated significant body weight change for the cohort. While a post hoc subgroup analysis of participants achieving ≥5% weight loss may be of interest, these findings should be identified as exploratory, non-generalisable, and not presented as a definitive intervention effect [5].

Further concerns relate to methodological clarity. Although six-month outcomes are presented, the statistical methods describe analyses of continuous outcomes from baseline to three months only, while six-month analyses are reported solely for categorical variables. Greater transparency regarding the analytic approach for six-month continuous outcomes would strengthen confidence in findings.

This issue is underscored by the near-identical three- and six-month outcomes reported for glycaemic and dietary measures (Table 1). In dietary intervention studies, outcomes typically change between timepoints, either through continued improvement or partial regression [6]. Such consistency warrants clarification regarding whether these represent independent analyses, reuse of data, or plateau effects.

Clarifying or correcting these points would enhance the scientific accuracy of the article, support appropriate comparison across studies, and ensure meaningful interpretation for both clinical and research purposes.

## Figures and Tables

**Table 1:** Kolivas et al. 3- and- 6-month outcomes comparison.

Outcome	6-Months [1]	3-Months [4]
HbA1c	−1.0%, 95% CI: −1.3 to −0.6	−1.0%, 95% CI: −1.3 to −0.7
Fasting glucose	−1.3 mmol/L, 95% CI: −2.0 to −0.6	−1.3 mmol/L, 95% CI: −2.1 to −0.6
Carbohydrate	−14% kJ/day, 95% CI: −17 to −11	−14% kJ/day, 95% CI: −17 to −11
Protein	6% kJ/day, 95% CI: −4 to −8	6% kJ/day, 95% CI: −4 to −7
Fat	9% kJ/day, 95% CI: 6 to 11	9% kJ/day, 95% CI: 6 to 11
